# Optimized Drop-Casted Polyaniline Thin Films for High-Sensitivity Electrochemical and Optical pH Sensors

**DOI:** 10.3390/polym16192789

**Published:** 2024-10-01

**Authors:** Bruna Eduarda Darolt Mücke, Beatriz Cotting Rossignatti, Luis Miguel Gomes Abegão, Martin Schwellberger Barbosa, Hugo José Nogueira Pedroza Dias Mello

**Affiliations:** 1 Physics of Materials Group, Institute of Physics, Federal University of Goiás, Samambaia Campus, Goiânia 74001-970, GO, Brazil; brunamucke@discente.ufg.br (B.E.D.M.); beatrizcotting@discente.ufg.br (B.C.R.); 2 Photonics Group, Physics Institute, Federal University of Goiás, Samambaia Campus, Goiânia 74001-970, GO, Brazil; luis.abegao@ufg.br; 3 Institute of Chemistry, Federal University of Goiás, Samambaia Campus, Goiânia 74001-970, GO, Brazil; martin_barbosa@ufg.br

**Keywords:** conducting polymer, drop-cast, chemical sensor, sensitivity

## Abstract

Conducting polymers used in chemical sensors are attractive because of their ability to confer reversible properties controlled by the doping/de-doping process. Polyaniline (PANI) is one of the most prominent materials used due to its ease of synthesis, tailored properties, and higher stability. Here, PANI thin films deposited by the drop-casting method on fluorine-doped tin oxide (FTO) substrates were used in electrochemical and optical sensors for pH measurement. The response of the devices was correlated with the deposition parameters; namely, the volume of deposition solution dropped on the substrate and the concentration of the solution, which was determined by the weight ratio of polymer to solvent. The characterisation of the samples aimed to determine the structure–property relationship of the films and showed that the chemical properties, oxidation states, and protonation level are similar for all samples, as concluded from the cyclic voltammetry and UV–VIS spectroscopic analysis. The sensing performance of the PANI film is correlated with its relative physical properties, thickness, and surface roughness. The highest electrochemical sensitivity obtained was 127.3 ± 6.2 mV/pH, twice the Nernst limit—the highest pH sensitivity reported to our knowledge—from the thicker and rougher sample. The highest optical sensitivity, 0.45 ± 0.05 1/pH, was obtained from a less rough sample, which is desirable as it reduces light scattering and sample oxidation. The results presented demonstrate the importance of understanding the structure–property relationship of materials for optimised sensors and their potential applications where high-sensitivity pH measurement is required.

## 1. Introduction

Polyaniline (PANI) is a conducting polymer (CP) that has a conjugated structure with delocalisation of π-electrons due to sp^2^ hybridised carbons in its backbone, which ensures its singular electrical and optical properties [1]. Its electrical, optical, and mechanical properties depend on the oxidation state of the polymer, which is defined by the ratio of amine to imine nitrogen atoms in the backbone. PANI can be found in three theoretical oxidation states: the fully reduced leucoemeraldine (PANI-LE), the fully oxidised pernigraniline (PANI-PE) and the half-reduced/half-oxidised emeraldine base (PANI-EB) [2]. Upon protonation of its imine nitrogen atoms, PANI-EB becomes emeraldine salt (PANI-ES), which is accompanied by changes in its morphology [3], colour [4], and conductivity [5]. The structural changes of PANI upon protonation (either chemically or electrochemically induced), combined with the possibility of tailoring its structure in the synthesis and deposition steps, make it useful for applications in science and technology, such as electronics, energy storage, biotechnology, and sensors [6,7,8,9,10,11,12]. An important class of chemical sensors is pH sensors. The measurement of pH is relevant in several industries and scientific fields. For example, wearable sensors for continuous biochemical monitoring [13]; nanomaterial-based devices for biomedical diagnostics [14]; colorimetric, photometric, fluorometric, conductometric, and piezoelectric sensors for soil pH detection in the agricultural field [15]; multi-parameter miniaturized, low-cost, and robust sensor chips for pH monitoring of water [16]; fibre optic-based pH sensors for monitoring the alkalinity of cementitious matrices in civil engineering [17]; and pH indicators immobilized into a hydrogel for oceanographic applications [18]. Therefore, the development of fast, accurate, and versatile pH sensors is of major interest to the advancement of those respective fields.

A chemical sensor is a device intended to convert the chemical information of test samples into analytical information. The primary device structure consists of a sensing stage that interacts with the target analyte and generates the signal to be converted by the transduction stage [19]. The materials used for the sensing stage in chemical sensors are usually required to have a large surface area and a fast sensor–target interaction that allow for a sensitive and fast response, both of which are met by PANI thin films when used for the detection of protons in aqueous solutions [20,21]. In the case of electrochemical and optical sensors, specialized deposition techniques are available for controlling the deposition of PANI over the substrate (electrode or optical window) [22].

High-quality sensors have been achieved by the simple, fast, and low-cost drop-casting method, resulting in sensitive thin films [23]. Zhao and co-workers obtained uniform reduced graphene oxide (RGO) films on poly(ethylene terephthalate) (PET) substrates using the drop-casting method [24]. The transparent and conductive films were evaluated for their sheet resistance and transmittance, which were found to be dependent on the deposition process. Wang and co-workers solved the limitations of organic photodetectors based on copper phthalocyanine films by applying the drop-casting method several times, which ensured a device with relatively high responsivity and low dark current [25]. Drop-casted PANI thin films stabilised with dinonylnaphthalene sulfonic acid (DNNSA) have been used in high-capacity electrochemical devices [26]. From the electrochemical characterisation, it was observed that specific capacitance improved due to the post-deposition processing and the deposition method allowing for a versatile substrate selection. Furthermore, drop-casting of PANI over screen-printed interdigitated electrodes (IDE) was performed for a conductometric pH sensor [27]. Compared to PANI-ES, the device worked correctly with PANI-EB, which showed an increased conductance with increasing pH. Hansen and co-workers obtained a PANI/poly(ethylene oxide) nanocomposite (PANI/PEO) with glucose oxidase samples by drop-casting and then depositing them over a platinum substrate for electrochemical glucose detection [28]. The device exhibited a reasonable linear range, sensitivity, and selectivity. Drop-casted PANI thin films are strong candidates for chemical sensing applications.

The optimal transduction system ensures an appropriate signal–to–noise ratio that affects the sensor’s detection limit with options such as electrochemical, optical, conductometric, and thermal platforms [29]. An optical pH sensor measures the change in the optical parameters of the sensing thin film after its exposure to the target analyte [30]. The most common optical parameters evaluated in optical sensors are visible light absorption (colorimetric sensors), fluorescence intensity and decay time, reflectance, refractive index, light scattering, and light polarisation [31]. Jin and co-workers measured the absorbance of PANI thin films prepared by oxidative polymerisation for a pH optical sensor with 1-month stability, with promising applications for colorimetric methods [32].

Electrochemical transducing systems are based on measurements performed in two- or three-electrode electrochemical cells that include the sensing stage as an electrode. The most common architecture for PANI-based pH sensors are potentiometric sensors, in which the concentration of H^+^ is measured through the open-circuit potential in equilibrium (E) and the concentration is obtained through the Nernst Equation:

(1) $$E=E_{0}^{\prime }-\frac{RT}{nF}\cdot \ln Q$$

in which $E_{0}^{\prime }$ is the formal electrode potential [33], R is the gas constant, T is the absolute temperature, F is Faraday’s constant, n is the number of moles of transferred electrons in the redox reaction, and Q is the reaction quotient. For pH sensors, the reaction quotient is defined as the ratio between the proton concentration on the film’s surface and the bulk electrolytes. The Nernst equation for pH measurement can be rewritten as:

(2) $$E=E_{0}^{\prime }-2.3\cdot \alpha \cdot \frac{RT}{F}\cdot pH$$

The α parameter depends on the ability of the sensing thin film to accumulate protons on its surface, given by the buffer capacity, and it is proportional to the number of surface active sites [34]. When α approaches 1, one can reach a maximum value of 59.2 mV/pH, the Nernst limit, for the sensitivity of a chemical pH sensor. Examples of PANI-based electrochemical pH sensors are the PANI nanofiber array-based pH sensors fabricated for the screen printing process [35], spin-coated and electrodeposited PANI thin films for potentiometric [36] and conductometric [37] pH monitoring, and PANI electropolymerized on a carbon fibre cloth (CFC) platform for pH measurements in food and pharmaceutical applications [38].

On the other hand, voltammetric sensors rely on dynamic current–potential voltammograms in which electrochemical currents or potentials associated with the redox peaks can be used as sensor signals. The Voltammetric approach can offer several advantages by allowing for better sensibility and higher signal–to–noise ratio, as its measurements are not limited by the equilibrium conditions required for potentiometric sensors [39]. In the case of PANI-based pH sensors, the shift in the redox potentials observed in different values of pH can be used as sensor signals [40], potentially resulting in sensitivities above the Nernstian limit [41,42,43]. Two fundamental parameters from the voltammetric curves can be used for the end: the cathodic peak potential ($E_{p}$) [42,43] or the half-wave potential, which is directly correlated with the formal potential ($E_{1/2}\approx E_{0}^{\prime }$) [44,45]. However, this approach is still underexplored compared to sensors based on potentiometric or current measurements. For instance, the effects of film thickness and morphology on the shift in voltammetric potentials can still be explored as a path for achieving more efficient sensors.

Therefore, this work sought to investigate the structure–property relationship in PANI thin film-based pH sensors. The roughness, thickness, and morphology parameters were controlled using the drop-casting method for sample preparation. The effects on the film fabrication in sensors were investigated using both colorimetric optical transduction and electrochemical voltammetric transduction.

## 2. Material and Methods

### 2.1. Materials

Fluorine-doped tin oxide (FTO) thin films deposited over a glass substrate (7 Ω/sq.), hydrous dibasic sodium phosphate (99%) and aniline were purchased from Sigma-Aldrich (St. Louis, MO, USA). Dimethyl sulfoxide (DMSO, 99%) and sulfuric acid (99%) were purchased from Merck (Whitehouse Station, NJ, USA), and citric acid (99.5%) was purchased from Vetec, Rio de Janeiro, Brazil. Hydrochloric acid (HCl, P.A.) was purchased from Neon, Sao Paulo, Brazil and ammonium persulfate (APS, reagent grade, 98%) from Synth, Brazil. Glass slides (KB7) were also used as the substrate for sample deposition. All of the chemicals were used as received without any further purification.

### 2.2. PANI Synthesis

PANI was synthesized via oxidative polymerization. The procedure was based on gradually adding 0.05 mol/L of aniline into 1.0 mol/L of HCl, previously cooled in an ice bath. Then, an aqueous solution of APS was slowly added drop by drop into the previous solution under continuous stirring at constant temperature in the ice bath for 4 h. The resulting material was then vacuum filtered and subjected to successive washings with distilled water, followed by air drying [46].

### 2.3. Sample Preparation and Structural Characterisation

The PANI thin films were deposited by the drop-casting method. FTO and glass thin films were used as substrates for sensing and characterisation after ultrasonic cleaning (DI-water, ethanol, and acetone, 20 min each). The PANI was deposited from a series of solutions with different weight ratios of polymer–solvent (DMSO): 1:100, 1:200, 1:300, 1:400 and 1:500. They were stirred and ultrasonicated for 2 h each before use. The volume of solution dropped onto the substrate was also controlled: 5, 10, 15 and 20 μL. The samples were dried in an oven at 60 °C for 30 min before being left to dry at room temperature, i.e., 298 K. The samples were labelled using both the volume of the solution deposited (XX) and information regarding the concentration of the solution (YYY) as tags, following the format of PANIXXYYY. For example, the sample deposited with a volume of deposition solution equal to 20 μL and a weight ratio of polymer–solvent equal to 1:500 was identified as PANI20500.

The thickness and average surface roughness of the samples were obtained using a DektakXT stylus (Bruker, Madison, WI, USA) profilometer. The scanning electron microscopy (SEM) characterisation was performed using a JEOL JSM—6610 system, with an operation voltage of 15–20 kV. Prior to the analysis, a 5 nm Au layer was sputtered over the samples to enhance the electrical conductivity. All experiments were performed at room temperature.

### 2.4. Electrochemical Proprieties and Electrochemical Sensor Characterizations

The electrochemical characterisation was performed using an AUTOLAB potentiostat (Metrohm, Herisau, Switzerland). The electrochemical setup contained a conventional three-electrode electrochemical cell system. The thin film samples were used as working electrodes. The reference electrode was an Ag|AgCl (3 mol/L) electrode, and platinum foil was used as the counter electrode. The cyclic voltammograms (CVs) were performed with a scan rate of 100 mV/s in a scan window between—0.2 to 1.2 V vs. Ag|AgCl in an aqueous sulfuric acid solution (0.5 mol/L). The electrochemical sensor measurements were performed using McIlvaine buffer solutions ranging from pH 2 to 8 as electrolytes. The buffer solutions were prepared using 0.1 mol/L of citric acid and 0.1 mol/L of sodium phosphate dibasic mixed in specific proportions.

The electrochemical sensor measurements followed the voltammetric approach, using the half-wave potentials (E_1/2_) obtained from the average potential between the oxidation and reduction peaks as a function of pH as the sensor signal [45], with the angular coefficient being the sensor sensitivity. The linearity was obtained from the coefficient of determination (R^2^) of the fitted calibration curves and was calculated as R^2^ times 100. The results are presented as a function of mean and standard deviation.

### 2.5. Optical Proprieties and Optical Sensor Characterizations

The UV–VIS absorbance spectra of the samples were recorded using a Lambda 1050 WB (PerkinElmer, Cambridge, MA, USA) spectrophotometer. The selected absorbance spectral window ranged from 350 to 850 nm with a 0.5 nm step. The optical sensor measurements were carried out in the spectrophotometer in the same configuration as described for sample characterisation with a 2 nm step. The PANI thin films were kept immersed in the buffer electrolytes for 60 s before the absorbance measurement. The films were carefully washed with distilled water between each measurement for both sensor setups. The sensitivity of the optical sensor was calculated from the sensor calibration curves. The linearity was obtained from the fitted calibration curves’ coefficient of determination (R^2^) and was calculated as R^2^ times 100.

## 3. Results and Discussion

### 3.1. Electrochemical and Spectroscopic Characterisation

**Figure 1:**
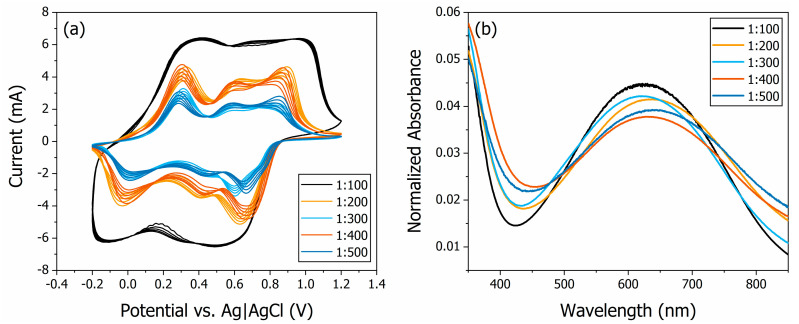
The electrochemical (**a**) and spectroscopic (**b**) characterisations of the set of PANI thin films deposited with a volume of deposition solution dropped on the substrate equal to 20 μL. The weight ratio of polymer–solvent for the samples is 1:100, 1:200, 1:300, 1:400 and 1:500.

The electrochemical and spectroscopic characterisations of the set of PANI thin films with a volume of deposition solution dropped on the substrate equal to 20 μL are shown in Figure 1. The cyclic voltammograms (CVs) of the films at a scan rate of 100 mV/s in 0.5 mol/L H_2_SO_4_ electrolytes are shown in Figure 1a. There are three redox pairs with oxidation peaks at approximately 0.30, 0.58, and 0.85 V vs. Ag|AgCl. The first and third redox pairs correspond to the changes in oxidation state of the PANI thin films: oxidation to emeraldine and oxidation to pernigraniline, respectively [47]. The appearance of an intermediate peak is a sign of possible degradation of the polymer over the cycles [48,49]. A higher peak current is observed for films prepared with a more concentrated deposition solution because the specific capacitance of the films is constant, and the injected charge is proportional to the mass of the polymer in the thin film.

The experimental UV–VIS absorption spectroscopy results of the films are shown in Figure 1b. The broad absorption peak in the 500 to 800 nm spectral region was observed for PANI in all its oxidation states and doping levels with slightly different positions. Nevertheless, both the lower energy band of the spectra, centred around 630 nm, and the higher energy band, centred around 350 nm, were assigned to the exciton transitions and to the π-π* benzenoid transition in the emeraldine base form of PANI. Also, the absorbance at around 420 nm should be absent in the emeraldine form of PANI and only present in the salt state of the polymer [50]. Qualitatively, the UV–VIS absorbance spectra of the PANI thin films appeared similar to each other, as previously discussed by Park and co-workers [51].

### 3.2. Electrochemical and Optical Sensors

**Figure 2:**
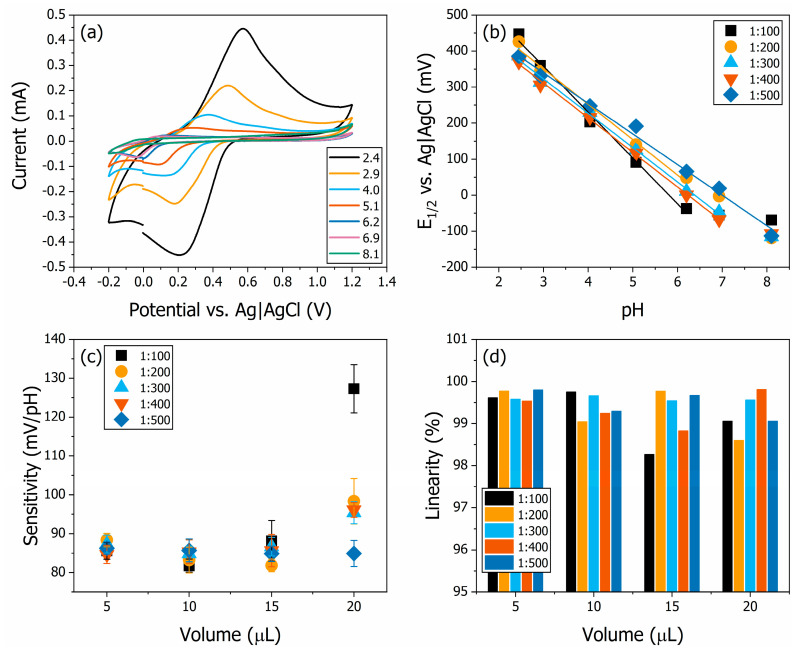
The electrochemical pH sensor for drop-casted PANI thin films. The CVs for different pH buffer electrolytes for sample PANI20500 are shown in (**a**). Calibration curves plotted as *E*_1/2_ vs. pH of the set of PANI thin films deposited with volume of deposition solution equal to 20 μL (**b**). The sensitivities of the samples are shown in (**c**). The sensitivities were calculated from the redox potential calibration curve, and the highest value obtained was 127.3 ± 6.2 mV/pH for sample PANI20100. The linearities of the samples are shown in (**d**). The lowest linearity obtained was 98.3% in PANI15100.

The electrochemical pH sensor measurements with the drop-casted PANI thin films are shown in Figure 2. The cyclic voltammograms (CVs) for the full range of pH buffer electrolytes for the sample PANI20500 are shown in Figure 2a. The interfacial processes occurring at the film surface depend on the pH of the electrolyte, which is an indicator of ion concentration. The protonation of the quinoid rings in PANI-based materials is responsible for the current measured via CV [5]. Therefore, decreasing the pH will increase the peak current. Also, the oxidation and reduction peaks are different for each pH, which is attributed to the electrostatic interaction between the ions and the polymer.

As expected from previous literature results, we observed that the anodic peaks shifted to more positive potentials as the concentration of H^+^ increased (lower pH). After extracting the values of *E*_1/2_ from the redox pairs, we observed a highly linear correlation between *E*_1/2_ and pH (Figure 2b). The dependency of the formal potential of the anodic voltammetric response of polyaniline in acidic solutions is discussed extensively by Marmisollé and co-workers. This behaviour differs from typical inert electrodes systems, in which the formal potential (and *E*_1/2_, by consequence) is not expected to shift with the concentration of the electroactive species in a solution. In the case of PANI/H^+^ interfaces, the formal potential is influenced by the degree of oxidation and the interaction energies between the redox sites in the polymeric matrix [44], allowing the voltametric approach for the calculation of the sensor signal.

The sensitivity and linearity values were extracted from the results presented in Figure 2b. The sensitivities of the samples are shown in Figure 2c. The sensitivities were calculated from the redox potential as a function of the pH calibration curves obtained for each sample. The highest sensitivity was obtained from sample PANI20100 at 127.3 ± 6.2 mV/pH. This sensitivity is more than twice the Nernst limit. All samples showed a super-Nernstian response, with the lowest sensitivity being 81.8 ± 1.7 mV/pH from PANI10100. There is a tendency for sensitivity to increase with an increasing volume of deposition solution. The highest variation between samples with different concentrations occurs in the 20 μL volume of deposition solution. The sensitivity ranges from 84.9 ± 3.4 mV/pH in PANI20500 to 127.3 ± 6.2 mV/pH in PANI20100. Increasing the deposition solution’s concentration increases the pH sensor’s sensitivity. Such a result indicates that all samples were functional as pH sensors. As mentioned, linearity was very high for all systems. The lowest value was observed in PANI15100 at 98.3%, and the majority of samples was higher than 99% (Figure 2d).

**Figure 3:**
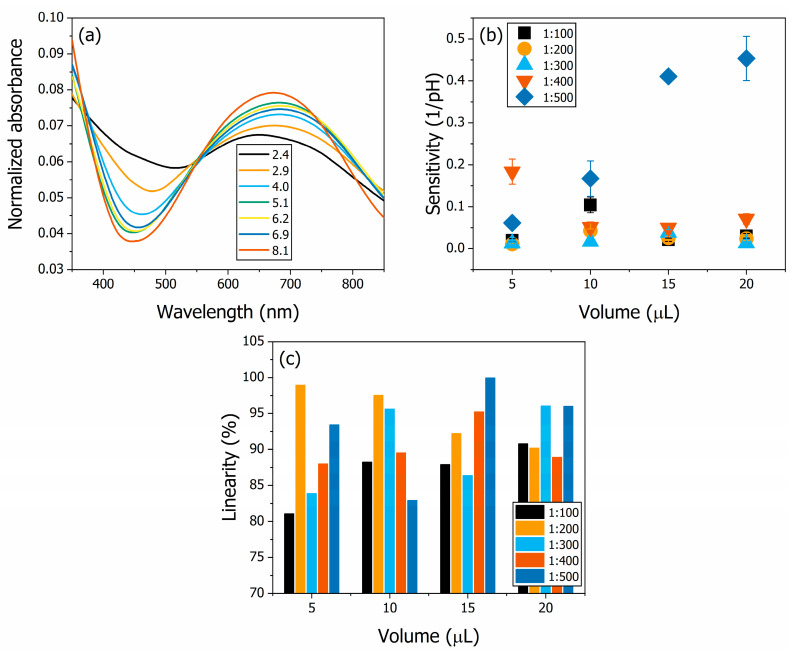
The optical pH sensor for drop-casted PANI thin films. The normalized absorbance spectra for different pH buffer electrolytes for sample PANI20500 are shown in (**a**). The sensitivities of the samples are shown in (**b**). The sensitivities were calculated from the calibration curve of integrated normalized absorbance, and the highest value obtained was 0.45 ± 0.05 1/pH in PANI20500. The linearities of the samples are shown in (**c**). The lowest linearity obtained was 81% in PANI05100.

The optical pH sensor measurements using the drop-casted PANI thin films are shown in Figure 3. The normalized absorbance spectra for the full range of pH buffer electrolytes in PANI20500 are shown in Figure 3a. Each curve shown is the absorption spectrum for a specific pH. The spectra show the same pattern of PANI-EB as observed in the characterisation (Figure 1b). The variation in the relative intensity of the absorption peaks is due to the protonation of the quinoid rings [52]. The sensitivities of the samples are shown in Figure 3b. The sensitivities were calculated from the calibration curves obtained for each sample. The calibration curves were plotted from the integrated normalized absorbance in the spectral region from 600 to 750 nm as a function of pH, as described elsewhere [53,54]. The highest sensitivity was obtained from sample PANI20500 at 0.45 ± 0.05 1/pH. The highest variation between samples with different concentrations occurred for the 20 μL volume of deposition solution in the electrochemical sensors. The sensitivity ranged from 0.03 ± 0.01 1/pH in PANI20100 to 0.45 ± 0.05 1/pH in PANI20500. Increasing the concentration of the deposition solution caused a decrease in the sensitivity of the optical pH sensor. The linearity of the samples is shown in Figure 3c. All samples showed a linearity higher than 81%, the value of film PANI05100, indicating that the samples could be safely used as optical pH sensors.

Since the electrochemical and spectroscopic characterisation of the PANI thin films indicates that all samples have the same chemical properties, oxidation states, and doping levels, the variations in the pH sensor response and the differences observed in the sensor response can be discussed in terms of thin film characteristics: namely, roughness, surface morphology, and thickness.

### 3.3. Thin Film Structural Characteriation

**Figure 4:**
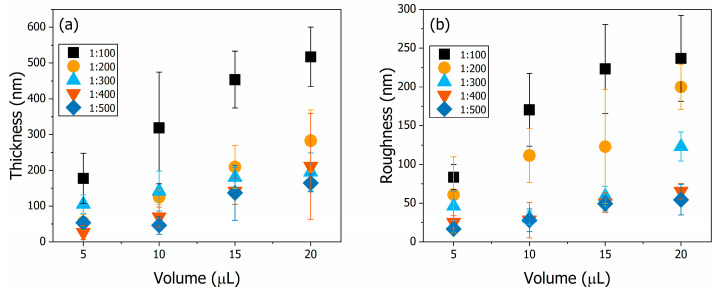
The thickness (**a**) and surface roughness, (**b**) R_Q_ (root mean square deviation) of the drop-casted PANI thin films. PANI20100 is the thicker and rougher sample.

The thickness and surface roughness R_Q_ (root mean square deviation) values obtained from the thin films by profilometry are shown in Figure 4. The thickness of each of the thin films is shown in Figure 4a. Increasing the volume used and the polymer concentration of the deposition solution caused an increase in the thickness of the films. PANI20100 was the thickest sample (517.4 ± 83.0 nm), as would be expected given that this is the PANI thin film that was produced with the highest volume and the most concentrated deposition solution. The thinnest film was sample PANI05400 (26.5 ± 9.6 nm). Quantitatively, the average surface roughness parameters for each of the thin films were calculated using the root mean square deviation and are shown in Figure 4b. Increasing the volume used and the polymer concentration of the deposition solution caused an increase in the surface roughness of the films. This left the PANI20100 sample as the roughest, with an average roughness of 236.9 ± 55.5 nm. The least rough film was sample PANI05500 (16.7 ± 3.8 nm). The highest thickness occurred for the set of PANI thin films produced with 20 μL of deposition solution, which had a positive effect on both sensors, as discussed previously.

**Figure 5:**
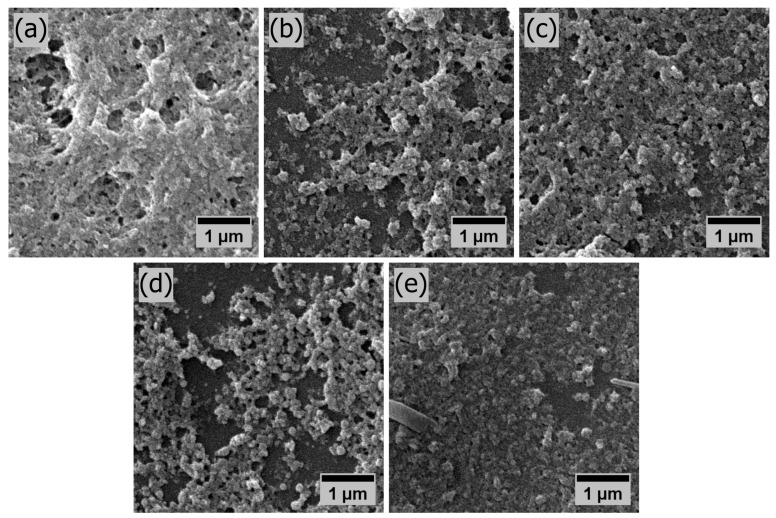
SEM characterization of the drop-casted PANI thin films deposited with 20 μL of solution. Samples deposited from solution with weight ratio: 1:100, 1:200, 1:300, 1:400 and 1:500 in (**a**–**e**), respectively.

Furthermore, the high roughness of the PANI20100 sample is favourable for electrochemical sensors with a potentiometric response, as the surface area of the film—correlated to its surface roughness—increases the sensor response by increasing the surface sites and, consequently, the charge at the electrolyte–film interface [55]. The lowest roughness among the films deposited with 20 μL of solution occurred in the PANI20500 sample. This is favourable for an optical sensor, as previously observed (Figure 3), since lower roughness reduces light scattering in combination with a smaller surface area, which reduces sample oxidation due to lower air–polymer interaction [30]. The morphology of the set of PANI thin films prepared with 20 μL of deposition solution is compared in the scanning electron microscopy (SEM) images shown in Figure 5. The samples prepared with decreasing solution concentration from 1:100 to 1:500 are shown in Figure 5a through 5e, respectively. Common to all samples is the specific nanostructured granular morphology, together with pores, of PANI deposited over the substrate, which became less dense with a decrease in solution concentration [56]. The smoothest surface of sample PANI20500 (Figure 5e) and the roughest surface of sample PANI20100 (Figure 5a) were consistent with the data presented in Figure 4.

**Figure 6:**
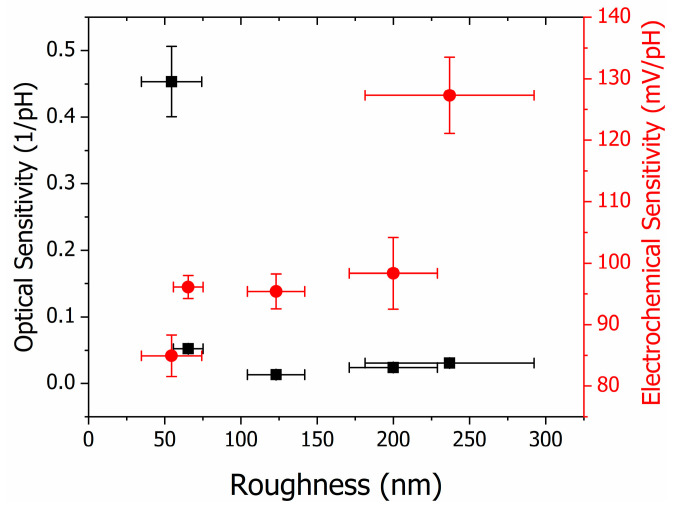
Optical (left *y*-axis) and electrochemical (right *y*-axis) sensitivity are shown according to the roughness of the PANI thin films deposited with 20 μL of solution.

The analysis of the sensitivity of the electrochemical and optical pH sensors as a function of sample roughness is shown in Figure 6. The inverse behaviour of the response of the electrochemical and optical sensors as a function of roughness was observed. The optical sensitivity showed a sharp decrease (0.45 ± 0.05 1/pH down to 0.05 ± 0.01 1/pH) for a small increase in roughness of 20% and remained approximately constant up to a roughness of 236.9 ± 55.5 nm. For the electrochemical sensors, an increase in roughness led to an increase in sensitivity. It started at 84.9 ± 3.4 mV/pH and increased approximately linearly up to 127.3 ± 6.2 mV/pH. By controlling the deposition parameters of the PANI thin films, it is possible to produce the optimal sample for each transducer platform.

Notably, the drop-casted PANI thin films showed a remarkable advantage in terms of higher sensitivity compared to other PANI films deposited by other techniques, particularly in electrochemical transduction systems, with values twice the Nernst limit observed. They were fabricated with a cost-effective method, although they presented a limited optical sensitivity and a narrower linear pH range, as shown in Table 1. Table 1 evaluates the performance of different electrochemical and optical pH sensors based on PANI films through a comparative analysis including deposition method, transduction platform, sensitivity, pH range, and linearity. The drop-casted PANI thin film (PANI20100) presented in this work showed the highest sensitivity (127.3 ± 6.2 mV/pH) for the electrochemical transduction platform, followed by the galvanostatic electrodeposited PANI thin film presented in the work of Mello and Mulato [54] with 81 ± 1 mV/pH. In general, the electrodeposition method presents samples with higher sensitivity than spin-coated samples. Only two PANI-based electrochemical sensors showed a sensitivity lower than the Nernst limit from the potentiometric sensors. This is an indication of the potential of PANI materials for super-Nernstian pH sensors. Due to the morphology of the final deposited samples, the electrodeposited PANI films showed higher optical sensitivity than the drop-coated films. It is important to note that the linearities of the electrochemical sensors were higher than those of the optical sensors. These results demonstrate the relevance of our research and its potential applications where high-sensitivity pH measurement is necessary. Once the structure–property relationship of PANI drop-casted thin films and the sensitivity and linearity of pH sensors are established, evaluating their performance in real-world applications and investigating their long-term stability and durability will be the subject of future work, as these aspects are critical for practical implementation.

## 4. Conclusions

The aim of the present study was to evaluate the effect of the drop-casting deposition method on the structure–property relationship of PANI thin films used in electrochemical and optical sensors for pH measurement. Although all samples presented similar chemical properties, oxidation states, and protonation levels as evaluated by voltametric and spectroscopic characterisation, the samples prepared with the highest volume of deposition solution (20 μL) and the highest concentration (defined as the weight ratio of polymer–solvent of 1:100), PANI20100 was the thickest, roughest and most heterogeneous. This sample showed the highest sensitivity of 127.3 ± 6.2 mV/pH using the shift in anodic peak as sensor signal, which is more than twice the Nernst limit from potentiometric sensors using the same interface. It was among the highest pH sensitivity results reported, to the best of our knowledge. The physical properties of the sample justify these results. As the roughest sample, PANI20100 had the highest surface area and an increased number of surface sites, which optimised the response of the sensor. The relationship between electrochemical pH sensor sensitivity and surface roughness was clear for all of the samples. Such a characteristic is not useful for an optical sensor, which was found by the results. Sample PANI20500 presented the highest sensitivity for an optical sensor at 0.45 ± 0.05 1/pH. This sample had the lowest roughness among the samples prepared with 20 μL of deposition solution, which reduces light scattering. In addition, it had a smaller surface area that reduced sample oxidation, which is critical for optical sensors. As surface roughness increased, the optical pH sensor sensitivity decreased. Control of the deposition process allows for the optimisation of the sensing layers of chemical sensors, and the results shown in this work demonstrate the potential relevance of drop-casted PANI thin films for applications where high-sensitivity pH measurement is required.

## Figures and Tables

**Table 1:** Comparison of PANI thin film-based electrochemical and optical pH sensors for the devices presented in this work and from the literature.

Deposition	Transduction	Sensitivity	pH Range	Linearity	Reference
Electrodeposition	Electrochemical	81 ± 1 mV/pH	2–8	99.9%	[54]
Electrodeposition	Electrochemical	70 ± 1 mV/pH	2–8	99.6%	[55]
Electrodeposition	Electrochemical	76 mV/pH	2–8	99.9%	[57]
Electrodeposition	Electrochemical	66 ± 1 mV/pH	2–8	99.0%	[58]
Electrodeposition	Electrochemical	74.2 ± 2.2 mV/pH	2–7	99.2%	[36]
Electrodeposition	Electrochemical	52 mV/pH	2–9	95.7%	[59]
Spin-coated	Electrochemical	56 ± 2 mV/pH	2–7	99.0%	[58]
Spin-coated	Electrochemical	62 ± 3 mV/pH	2–8	98.7%	[36]
Drop-casted	Electrochemical	127.3 ± 6.2 mV/pH	2–6	99.1%	This work
Chem. oxidation	Optical	0.09 1/pH	5–8	-	[32]
Electrodeposition	Optical	25 1/pH	2–8	97.4%	[53]
Electrodeposition	Optical	30 ± 3 1/pH	2–6	92.0%	[54]
Drop-casted	Optical	0.45 ± 0.05 1/pH	2–7	95.2%	This work

## Data Availability

The original contributions presented in the study are included in the article, further inquiries can be directed to the corresponding author.
